# Secondary Cartilage Revealed in a Non-Avian Dinosaur Embryo

**DOI:** 10.1371/journal.pone.0056937

**Published:** 2013-02-13

**Authors:** Alida M. Bailleul, Brian K. Hall, John R. Horner

**Affiliations:** 1 Museum of the Rockies, Montana State University, Bozeman, Montana, United States of America; 2 Department of Earth Sciences, Montana State University, Bozeman, Montana, United States of America; 3 Department of Biology, Dalhousie University, Halifax, Nova Scotia, Canada; University of Pennsylvania, United States Of Amerca

## Abstract

The skull and jaws of extant birds possess secondary cartilage, a tissue that arises after bone formation during embryonic development at articulations, ligamentous and muscular insertions. Using histological analysis, we discovered secondary cartilage in a non-avian dinosaur embryo, *Hypacrosaurus stebingeri* (Ornithischia, Lambeosaurinae). This finding extends our previous report of secondary cartilage in post-hatching specimens of the same dinosaur species. It provides the first information on the ontogeny of avian and dinosaurian secondary cartilages, and further stresses their developmental similarities. Secondary cartilage was found in an embryonic dentary within a tooth socket where it is hypothesized to have arisen due to mechanical stresses generated during tooth formation. Two patterns were discerned: secondary cartilage is more restricted in location in this *Hypacrosaurus* embryo, than it is in *Hypacrosaurus* post-hatchlings; secondary cartilage occurs at far more sites in bird embryos and nestlings than in *Hypacrosaurus.* This suggests an increase in the number of sites of secondary cartilage during the evolution of birds. We hypothesize that secondary cartilage provided advantages in the fine manipulation of food and was selected over other types of tissues/articulations during the evolution of the highly specialized avian beak from the jaws of their dinosaurian ancestors.

## Introduction

The skulls and jaws of extant birds possess a specific type of cartilage known as *secondary cartilage* (SC), so named because it arises secondarily on pre-existing membrane bones [1]–[10] and not before bone (primarily), as does the primary cartilage model of endochondral bones (see [11]). SC arises during embryonic and early post-hatching development as an articular cartilage at joints or as nodules associated with ligamentous or muscular insertions, in all cases arising in response to intermittent pressure and shear [2]–[4]. SC forms because of the ability of the ‘osteogenic’ periosteal precursor cells to form chondroblasts in addition to osteoblasts [9]. This chondrogenic potential of the periosteum — the presence of osteochondroprogenitor cells [4], [5] — is only found in birds among living sauropsids and lissamphibians, which do not form SC but rather accommodate stress and strain with syndesmoses, i.e., the deposition of dense networks of collagen fibers at articulations [12]–[15]. Outside sauropsids and lissamphibians, a tissue known as SC is found in mammals and teleosts [16]–[19]. Due to their phylogenetic distribution, teleostean, mammalian and avian SCs are hypothesized to be homoplastic ([9], see [20] for further details). A notable difference is that mechanical stimulation is not required for the initiation of mammalian SCs [17], while it is needed for the formation of avian SCs [9].

**Figure 1:**
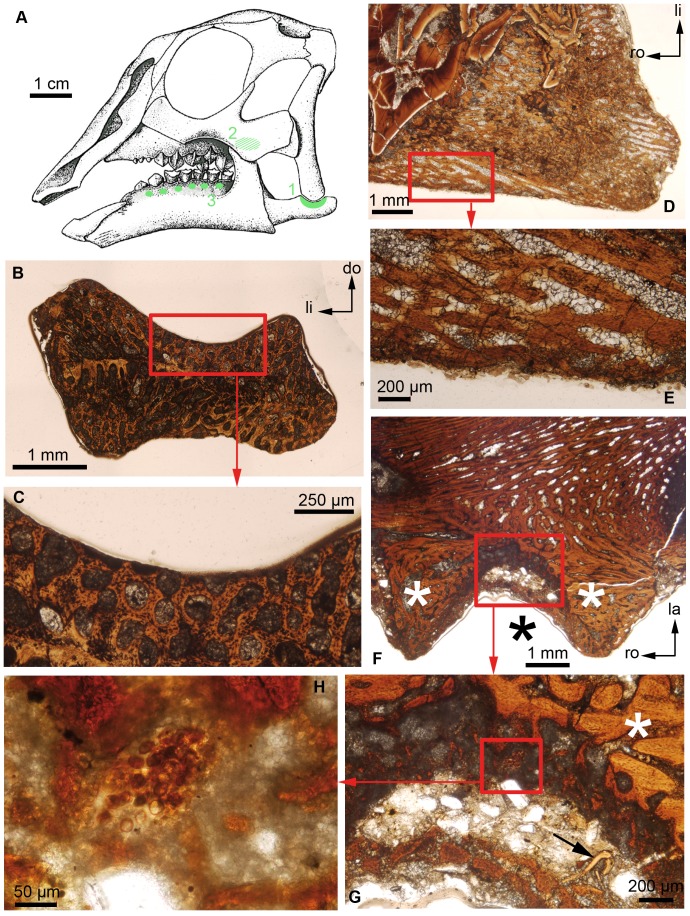
Secondary chondrogenesis investigated in hadrosaurid embryos. (**A**) Reconstruction of the embryonic skull of *Hypacrosaurus stebingeri,* reproduced with permission [21] with anatomical locations 1, 2 and 3 in green. (**B**) Transverse section of the surangular of a Hadrosauridae indet. (MOR 1038). (**C**) Close-up of the red box in (B). The dorso-caudal face (Location 1) does not show any remnant of SC. (**D**) Coronal section of the maxilla of *Hypacrosaurus stebingeri* (MOR 559). (**E**) Close-up of the red box in (D). The bucco-caudal face of the maxilla (Location 2) does not show any remnants of SC. (**F**) Coronal section of the dentary of *Hypacrosaurus stebingeri* (MOR 559). (**G**) Close-up of the red box in (F). The arrow indicates a remnant of dentine. (**H**) Close-up of the red box in (G). (F) and (G) show alveolar bone (white asterisks) and incomplete alveoli with missing teeth (black asterisk; Location 3). (G) and (H) show a SC islet. All sections are shown under natural light. do, dorsal; la, labial; li, lingual; ro, rostral.

In a previous study [20], we reported for the first time the presence of SC in a non-avian dinosaur, *Hypacrosaurus stebingeri* (Ornithischia, Lambeosaurinae), and hypothesized that it was homologous to “avian” SC; see [20] for the criteria used to establish homology of avian and dinosaurian SC. That study [20] focused on post-hatching specimens. To investigate secondary chondrogenesis through ontogeny and to compare the avian and ornithischian patterns it was necessary to investigate non-avian dinosaur embryos. In the present study using histological analyses of isolated skull elements of embryonic *H. stebingeri* (Figure 1A), *Maiasaura peeblesorum* (Hadrosaurinae) and a Hadrosauridae indet., we report for the first time the presence of SC in a non-avian dinosaur embryo. From our analyses we propose that different patterns of the onset of secondary chondrogenesis exist in ornithischian and saurischian (avian) embryos, that these different patterns reflect different biomechanical conditions within avian and dinosaurian embryos that may be explained by the evolution of the highly specialized avian beak from the jaws of their dinosaurian ancestors.

## Materials and Methods

### Ethics

All necessary permits were obtained for the described field studies. Permission for the collection of MOR 1038 was granted by the Montana Department of Natural Resources (Helena, MT). The other MOR specimens were collected with land owner permission from private land (and therefore do not recquire any specific permit).

The six embryonic skull bones examined in this study (Table 1) were all disarticulated and collected from nesting horizons in the Two Medicine (TM) and Judith River (JR) Formations (Upper Cretaceous) in Montana. They belong to three closely related species: *Hypacrosaurus stebingeri*, *Maiasaura peeblesorum* (Hadrosaurinae) and a Hadrosauridae indet. The *Hypacrosaurus* specimens MOR 559 come from a nesting ground located on Blacktail Creek, Glacier County (Locality TM-066; see [21]) and these embryonic bones were weathering out of the eggs of a clutch. This is the very same locality that yielded the numerous post-hatching specimens MOR 548 used in our previous study [20]. The embryonic *Maiasaura* MOR-YPM.430.Sa was collected in the Willow Creek Anticline, in a nesting ground near Choteau, Teton County (Locality TM-160; see [22], [23]). This bone was associated with its egg. Finally, the Hadrosauridae indet., was also collected from a nesting ground located North of Kremlin, Hill County (Locality JR-144Q). Table 2 shows all anatomical locations where secondary chondrogenesis was sought (in priority, the three previously known cartilaginous sites [20], but also any other sites potentially subject to pressure and shear). Archival molds and casts were made prior to sectioning. The disarticulated bones were embedded in epoxy resin and cut with a diamond powder disk on a precision saw. All elements were serially thin-sectioned (except for the parietal and frontal), mounted on plastic slides and then ground and polished by hand on a Buehler Ecomet grinder. Finished thin-sections were studied by light microscopy under normal and polarized light.

**Table 1:** List of the hadrosaurid membrane bones thin-sectioned and examined in this study.

Taxon	Specimen no.	Ontogenetic stage	Element	Length (rosto-caudal) (cm)	Formation and locality
Hadrosauridae indet.	MOR 1038	embryonic	Surangular	2.1	Judith River: JR-144Q
*Hypacrosaurus stebingeri*	MOR 559	embryonic	Dentary*	5.1	Two Medicine: TM-066
*Hypacrosaurus stebingeri*	MOR 559	embryonic	Frontal	3.6	Two Medicine: TM-066
*Hypacrosaurus stebingeri*	MOR 559	embryonic	Maxilla	5.0	Two Medicine: TM-066
*Hypacrosaurus stebingeri*	MOR 559	embryonic	Parietal	3.2	Two Medicine: TM-066
*Maiasaura peeblesorum*	MOR-YMP430.Sa	embryonic	Surangular	1.7	Two Medicine: TM-160
*Hypacrosaurus stebingeri*	MOR 548	post-hatching (in [20])	Dentary*	9.0	Two Medicine: TM-066
*Hypacrosaurus stebingeri*	MOR 548	post-hatching (in [20])	Maxilla*	8.8	Two Medicine: TM-066
*Hypacrosaurus stebingeri*	MOR 548	post-hatching (in [20])	Surangular*	4.1	Two Medicine: TM-066

(The asterisks designate bones that had remnants of SC)

**Table 2:** List of the sites analysed for secondary chondrogenesis.

Articulations or contact zones
dentary-predentary
dentary-surangular
mandibular symphysis
maxilla-coronoid process of dentary (Location 2)
maxilla-jugal
maxilla-premaxilla
maxilla-pterygoid
surangular-angular
surangular-quadrate (Location 1)
**Alveolar processes**
dentary* (Location 3)
Maxilla
**Muscle insertion sites ( see [35])**
m. pterygoideus ventralis (surangular)
m. pseudotemporalis profundus (surangular/mandibular fossa)
m. pseudotemporalis superficialis (coronoid process/mandibular fossa)
m. adductor mandibulae externus profundus (coronoid process)
m. adductor mandibulae externus medialis (coronoid process/surangular)
m. adductor mandibulae externus superficialis (surangular)
m. adductor mandibulae posterior (mandibular fossa)
m. depressor mandibulae (surangular)
**Sutural areas**
frontal-frontal
frontal-nasal
frontal-parietal
parietal-frontal
parietal-squamosal

(The asterisk indicates where SC was found).

## Results

**Figure 2:**
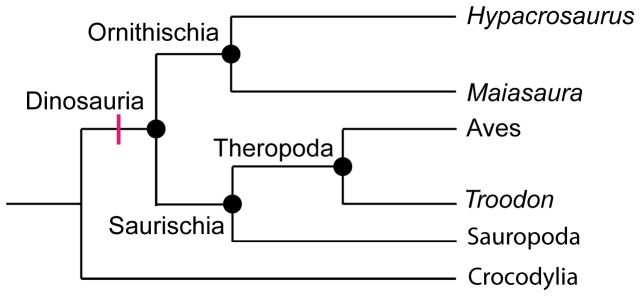
Phylogenetic relationships of some dinosaurian species and clades. Cladistic analysis (e.g., [34]) divides the Dinosauria into the Ornithischia (including *Hypacrosaurus* and *Maiasaura* from this study) and the Saurischia (with the Sauropoda and the Theropoda, the latter including birds). Based on the distribution of secondary cartilages discussed in this paper, secondary chondrogenesis is hypothesized to be a synapomorphy of the Dinosauria (red dash).

Since SC arises during embryonic development [2] and persists after hatching in extant birds [4], [5] it was necessary to investigate in priority the same anatomical locations that showed SC in the *H. stebingeri* post-hatchlings [20]. These are (1) the dorso-caudal face of the surangular (at its articulation with the quadrate; Figure 1A,B,C); (2) the bucco-caudal face of the maxilla (directly facing the coronoid process of the dentary; Figure 1A,D,E); and (3) alveolar spaces between dentary teeth (Figure 1A,F–H).

As a result of this survey, a single SC islet was found in location 3 within a tooth socket in the embryonic dentary of *H. stebingeri* (Figure 1A,F–H). This islet is unique in our sample, as no SC was present on localities 1 or 2 (Figure 1A,C,D), nor on any of the other sites investigated (Table 2).

This islet is composed of large round cells, typical of hypertrophied chondrocytes (embedded in a sparse extracellular matrix) and differs from the surrounding flattened osteocytes in the alveolar bone (Figure 1H). A small layer of dentine is present in the immediate vicinity of the cartilaginous islet (Figure 1G). As interpreted for the SC islets found between the dentary teeth of the *H*. *stebingeri* post-hatchlings [20] this islet is hypothesized to have arisen due to the stress generated by odontogenesis [24], [25]. Such SCs had already been found in the alveolar processes of the human dentary and maxilla by Masquelin [26] and Schaffer (see review [7]) over 130 years ago. The possible confusion of this cartilage with Meckel’s cartilage (MC) was discussed extensively in a study on birds [2]. MC is unlikely to be found in a tooth socket; it is localized more ventrally within the dentary in vertebrates. Moreover, in all the serial thin-sections, no remnant of MC was found, the Meckelian groove being entirely filled with calcite. This suggests that MC was made of unmineralized hyaline cartilage at that stage (it remains as a permanent cartilaginous rod in most extant taxa, [11]), and that the alveolar cartilage is indeed SC.

## Discussion

In this study, we report for the first time the presence of SC in a non-avian dinosaur embryo, the ornithischian *H. stebingeri* (Figure 1H). This supports the conclusions of our previous study in which SC was found in post-hatching skulls of the same species [20]. The combined results of our two studies show the distribution and persistence of secondary chondrogenesis through two ontogenetic stages of one non-avian dinosaur species. This distribution can now be compared to the patterns observed in extant birds [2], [4], [5].

First, that SC was found in an embryo, and second, that this site persists after hatching [20] (as it does in extant birds [2], [4], [5]) provides further support for the similarity of avian and ornithischian SC. As we previously hypothesized [20], this strongly suggests that secondary chondrogenesis (or the chondrogenic potential of the periosteum) is a synapomorphy for the clade containing the last common ancestor of *H. stebingeri* and extant birds, with all its descendants, i.e., the Dinosauria ([27] and Figure 2). Indeed, even though the condition in non-avian saurischians is still unknown, it is more plausible to assume that this complex ability of the periosteum (to switch from osteogenesis to chondrogenesis) evolved once in all the Dinosauria, rather than twice and independently in the Ornithischia and the Aves [20].

Before we give a paleobiological interpretation to our findings, it is important to take taphonomy into account. It may be that additional mineralized SC nodules existed at this embryonic stage but were removed by some post-mortem process without leaving a trace of their former presence (e.g. see [28]). However, given the excellent microstructural preservation, and the absence of abrasion on the edges of the bones (where SC would be found if it were present), our biological interpretation is that the SCs of locations 1 and 2 had not yet arisen (Figure 1C,E), or had arisen but had not yet calcified.

In chick embryos, SCs arise halfway through development (on the 11^th^ day out of 21 days of incubation) and is calcified in part much later (by the 15^th^ day) [6]. The size of *Hypacrosaurus* eggs, with diameters between 18 and 22 cm [23], suggests a much longer incubation time for *Hypacrosaurus* than for domestic chickens. Among birds, larger eggs correlate with longer incubation periods [29], which would give ample time for new SC sites to arise and calcify.

Based on the volume of an average *Hypacrosaurus* egg (estimated at 3900 ml; [21]), we calculated a weight of 4251g (see [30] for the formula) and estimated the incubation time as 74 days (based on the relationship determined by Rahn and Ar [31] linking incubation time and egg weight). Moreover, the size (see rostro-caudal length in Table 1) of the embryonic bones of these specimens and a comparison with the skeletal reconstructions in Horner and Currie [21] suggests that the embryos were between 2/3 and 3/4 of the way through embryonic development, leaving approximately 20 to 25 days left until hatching, which is much more time than that available to chicken embryos to calcify their nine SC centers (i.e. 6 days). Nevertheless, we found that SC was extremely rare in our sample of ornithischian embryos, with only one SC islet present in a dentary. Later in ontogeny, the number of SC nodules increases (with three sites after hatching [20]), but the number of sites is still lower than that observed in bird hatchlings (seven sites for nestling chicks [5]). Although, the limited sample size of the embryonic material in this study does not allow any definitive conclusion or generalization, these preliminary results do suggest that the relative abundance (and contribution) of SC differs during the normal development of the skull of extant birds and non-avian dinosaurs.

These different patterns of secondary chondrogenesis through ontogeny could be explained by either of two hypotheses, one developmental, one evolutionary. First, a difference in the embryonic motility of the avian and ornithischian embryos could be considered, with a lower motility and/or a higher threshold for the initiation of secondary chondrogenesis in ornithischian embryos. Indeed, movement during the embryogenesis of the chick is necessary for the normal development of SC [8]. If chick embryos are paralyzed, SC does not form in the skull, but does on the clavicle (the only post-cranial membrane bone) because of the passive movements of the amnion (i.e., clavicle SC requires less movement to be initiated than do skull SCs [8]).

Second, the increase in the number of sites of SC in the bird lineage could be linked to the evolution of the highly specialized avian beak. A factor responsible for this increase could be mainly mechanical, i.e., intermittent pressure and shear within the beak, increasing during evolution (and possibly different from the forces acting on the dinosaurian jaws). For example it is known that the black skimmer, *Rynchops niger,* that flies with its lower jaw in the water (with tremendous pressure acting on it), possesses much larger amount of SC than birds with other lifestyles [1]. A study focusing on SC in relation to lifestyle in birds of various sizes and feeding adaptations should shed light on the functional significance of secondary chondrogenesis. Moreover, the increase in the number of sites of SC in the bird lineage must have been under selection as joints, articulations, muscular and ligamentous insertions originated. Indeed, during the evolution of the bird beak, with all its numerous and diverse adaptations (e.g., [32]), SC could have provided advantages in the fine manipulation of food (e.g., [33]), and been selected over other types of tissues/articulations. Again, these functional hypotheses are preliminary considering the small sample size of our study (and the unknown condition in non-avian theropods). Nevertheless, this study does suggest that a change in the pattern of secondary chondrogenesis was an important step during the evolution of the avian beak from dinosaurian jaws.
